# Bone Health Management in Men Commencing Androgen Deprivation Therapy for Prostate Cancer and Women Commencing Anti‐Oestrogen Therapy for Breast Cancer

**DOI:** 10.1002/cam4.70873

**Published:** 2025-05-13

**Authors:** Ian Liang, Sarah Brennan, Christian Girgis, Amy Hayden, Tania Moujaber, Sandra Turner, Anuradha Vasista, Mathis Grossmann, Peter K. K. Wong

**Affiliations:** ^1^ Institute of Rheumatology and Orthopaedics Royal Prince Alfred Hospital Camperdown, Sydney New South Wales Australia; ^2^ Faculty of Medicine and Health University of Sydney Camperdown New South Wales Australia; ^3^ Faculty of Medicine Western Sydney University Campbelltown New South Wales Australia; ^4^ Department of Endocrinology Westmead Hospital Westmead New South Wales Australia; ^5^ Crown Princess Mary Cancer Centre, Westmead Hospital Westmead New South Wales Australia; ^6^ Department of Endocrinology Austin Health Heidelberg Victoria Australia; ^7^ Department of Medicine University of Melbourne Carlton Victoria Australia; ^8^ Department of Rheumatology Westmead Hospital Sydney New South Wales Australia

**Keywords:** androgen deprivation therapy, aromatase inhibitor, bone health, breast cancer, fracture prevention, osteoporosis, prostate cancer

## Abstract

**Background:**

Survival of patients with prostate and breast cancer, the commonest cancer in men and women, respectively, has markedly improved with advances in early diagnosis, treatment and multi‐disciplinary care by the oncology and surgical community. However, the use of increasingly potent endocrine therapies may cause bone loss, resulting in secondary osteoporosis.

**Review:**

This review summarises the current management of cancer treatment‐induced bone loss in this group of patients at high risk of osteoporotic fractures with their attendant morbidity and mortality.

**Conclusion:**

Bone health is an increasingly important part of cancer survivorship. Radiation and medical oncologists, urologists, bone health experts, general practitioners, healthcare professional bodies and bone health and cancer consumer organisations should increase awareness of the potential adverse effect of endocrine therapy on bone health. While this should never delay cancer treatment, bone health should be part of routine care for men and women receiving endocrine therapy for prostate and breast cancer.

## 1 Introduction

Survival of patients with prostate and breast cancer has markedly improved with advances in treatment, such as endocrine therapies. However, these therapies may cause bone loss, resulting in secondary osteoporosis. This review will summarise the current management of cancer treatment‐induced bone loss (CTIBL) in this high‐risk group of patients.

## 2 Literature Search Strategy for Prostate and Breast Cancer

### 2.1 Data Sources and Searches

A systematic review was conducted according to Preferred Reporting Items for Systemic Reviews and Meta‐Analyses (PRISMA) Guidelines. Ovid MEDLINE (1996 to 08 May 2024), EMBASE (1996 to 08 May 2024) and PubMed (08 May 2024) were searched from 2000 to 2023 with the following terms: “prostate cancer” and “androgen deprivation,” “breast cancer” and “anti‐oestrogen therapy,” “bone protective therapy” and “osteoporosis.” Filters were applied to limit results to studies published in English and to randomised controlled trials (RCTs). IL, SB and PW reviewed titles and abstracts for prostate cancer and breast cancer, followed by a detailed review of full texts.

### 2.2 Study Selection

English‐language RCTs were included if the following criteria were met: (i) the study population consisted of men with prostate cancer treated with androgen deprivation therapy (ADT) or women with breast cancer treated with anti‐oestrogen therapy; (ii) the intervention involved bone protective drug therapies directed at improving bone health (improved bone mineral density [BMD] or bone density and fracture prevention) in patients with non‐metastatic disease; and (iii) the intervention was compared to placebo or other pharmacologic agents.

### 2.3 Results

The search for the broad topic of bone health in prostate cancer identified 69 results from PubMed, 25 results from Ovid MEDLINE (1996 to 26 April 2024) and 35 results from EMBASE (1996 to 26 April 2024)—resulting in a total of 129 records. Of these, 88 records were excluded because they were identical papers or not relevant based on title and abstract. A total of 41 trials were assessed and 14 excluded because they did not fit selection criteria as outlined above. Overall, 27 RCTs met inclusion criteria. A similar search of bone health in breast cancer identified 145 results from PubMed, 170 from Ovid MEDLINE (1996 to 26 April 2024) and 326 from EMBASE (1996 to 26 April 2024), totalling 641 records. After excluding 576 records for being identical papers or irrelevant based on title and abstract, 65 trials were further evaluated. Of these, 36 were excluded for not meeting the selection criteria. Ultimately, 29 RCTs were included in the review.

## 3 Prostate Cancer

### 3.1 Osteoporosis and Androgen Deprivation Therapy

Prostate cancer is the commonest cancer affecting men, with one in six men diagnosed before the age of 85 years [1]. Multi‐modal treatment, including ADT—used in almost half of men with prostate cancer—has led to 5‐year survival rates of over 92% [1]. However, because it reduces serum testosterone and its aromatisation product, estradiol to castrate levels, ADT is associated with CTIBL, resulting in decreased bone mineral density (BMD) [2, 3]. This may be accentuated by newer treatments used for prostate cancer, such as glucocorticoids, androgen receptor pathway inhibitors (ARPIs) and radiation therapy.

In healthy men, BMD decreases by 0.5%–1% per year starting in mid‐life. However, ADT accelerates this to 3%–5% per year, even without skeletal metastases [4]. The BMD reduction occurs early, with loss of 2.5% at the hip and 4.0% at the lumbar spine in the first 12 months following ADT initiation [2, 3].

In non‐metastatic prostate cancer, osteoporosis (dual‐energy x‐ray absorptiometry; DXA *T*‐score ≤ −2.5, ie 2.5 or more standard deviations below young normal controls), was identified in 35.4% of ADT‐naïve men, 42.9% after 2 years of ADT and 80.6% after ≥ 10 years of ADT [5]. In Australia, approximately half the men with prostate cancer had low BMD even before ADT initiation [6]. Furthermore, ADT initiation is often superimposed on the inflexion point of the age‐dependent rise in fragility fractures [7].

The risk of fractures in men with prostate cancer receiving ADT has been extensively studied. A large meta‐analysis of 16 studies involving 519 168 men with prostate cancer found that ADT use was significantly associated with increased fracture risk (odds ratio; OR 1.39; 95% confidence intervals; CI, 1.26–1.52) and fractures requiring hospitalisation (OR 1.55; 95% CI, 1.29–1.88) [8]. Osteoporotic fractures are associated with increased morbidity and mortality [9, 10]. Furthermore, the addition of ARPIs increases the risk of both fractures and falls compared with ADT alone, further complicating bone health management in these patients [11, 12].

### 3.2 Diagnosis of Cancer Treatment‐Induced Bone Loss (CTIBL)

Bone density is usually assessed using DXA measurements at the hip and spine. Clinical risk prediction tools such as the Fracture Risk Assessment Tool (FRAX) may be helpful for estimation of absolute fracture risk [13]. While FRAX estimates the 10‐year absolute risk of an osteoporotic fracture based on risk factors such as age, gender, weight, height, family history and secondary osteoporosis (including hypogonadism), it has, until recently, not been validated in men with prostate cancer. However, a recent study from the Manitoba BMD Registry reported that FRAX reliably predicted incident fractures in men with prostate cancer [14].

Multiple guidelines recommend DXA assessment at the time of ADT initiation [15, 16, 17]. While single centres may have excellent DXA screening [18], only 20% of Australian men in the 6 months prior to or within 12 months following commencement of ADT for prostate cancer were referred for a DXA scan [19]. In North America, only 8.6% of men with prostate cancer had DXA scanning within the 12 months prior to or 6 months following commencement of ADT [20]. Similarly, only 19.4% of Indian urologists routinely performed a baseline DXA before starting ADT [21], with similar findings in Scotland [22]. Barriers to DXA assessment included lack of time, poor supporting clinical practice infrastructure, cost and lack of government subsidy [23]. These factors, along with the possible underestimation of the deleterious effect of ADT on skeletal health and the impact of fragility fractures on morbidity and mortality, may have also contributed.

### 3.3 Non‐Pharmacological Treatment of CTIBL

It is generally recommended that patients with prostate cancer engage in regular exercise for beneficial effects on excess adiposity and prevention of sarcopenia—common adverse effects of ADT [24]. However, no clear evidence exists that exercise prevents bone loss and reduces fracture risk in patients with prostate cancer [25]. Other recommended lifestyle interventions as per the European Society for Medical Oncology (ESMO) include smoking cessation, safe alcohol intake and maintenance of a healthy weight [26].

### 3.4 Vitamin D and Calcium Supplementation

The United States Preventive Services Task Force has recommended against routine calcium and vitamin D supplementation in non‐institutionalised older people [27]. However, there is reasonable evidence of benefit for those who may be deficient, particularly institutionalised individuals or frail older people, including those with cancer [28]. The National Comprehensive Cancer Network (NCCN) recommends oral supplementation of vitamin D (800–1000 IU/day) if deficient and an adequate calcium intake (1000–1200 mg/day) [29]. A similar recommendation is made by the Royal Australian College of General Practitioners and Healthy Bones Australia [30]. This is sensible advice as all participants in randomised controlled intervention trials of bone protective agents were routinely treated with calcium and vitamin D supplements [31, 32, 33, 34, 35].

### 3.5 Bone Protective Agents

Bone protective therapy can be divided into:

- *anti‐resorptive agents*, such as bisphosphonates and denosumab, which inhibit bone resorption; and
- *bone anabolic agents*, such as romosozumab and parathyroid hormone (PTH) analogues, which predominantly stimulate bone formation.

Randomised controlled trials of bone protective therapy in this population have mostly used bisphosphonates (Table 1)—zoledronic acid [31, 32, 33, 38, 39, 40, 41, 42, 43, 44, 45, 46, 47] pamidronate [36, 37] alendronate [34, 53] risedronate [51, 52] or denosumab [35, 48, 49] (Table 1 shows these arranged by name). While these studies reported increased BMD compared to placebo, they were not large enough to assess fracture rates. Denosumab has so far been the only bone protective agent associated with reduced fracture risk—albeit as a secondary endpoint [35].

**TABLE 1:** Randomised controlled trials of bone protective therapy in patients with prostate cancer treated with androgen deprivation therapy.

Reference number	ADT used	Inclusion criteria	Drug	Dose	BMD change in intervention group	Control	BMD change in controls	Patient numbers	Timepoint of primary outcome
Diamond 2001 [36]	Goserelin + flutamide or bicalutamide	No BMD criteria	APD	90 mg IV once (crossover study at 6 months)	FN +2%	PBO	FN −2.3%	18 men	6 months
Smith 2001 [37]	Leuprolide	No BMD criteria	APD	60 mg IV every 3 months	LS +0.5% TH +0.2%	PBO	LS −3.3%, −1.8% TH	21 APD/22 PBO	48 weeks
Smith 2003 [31]	GnRH agonist +/− an antiandrogen	No BMD criteria. Excl if *T*‐score < −3	ZOL	4 mg IV every 3/12	LS +5.6%, TH +1.1%	PBO	LS −2.2%, TH −2.8%	55 ZLD/51 PBO	12 months
Ryan 2006 [38]	GnRH agonist or orchiectomy	No BMD criteria. Excl if *T*‐score < −2.5	ZOL	4 mg IV every 3/12	LS +4.6%, FN +1.3%	PBO	LS −2.1%, FN −2.4%	61 ZOL/59 PBO	12 months
Israeli 2007 [39]	GnRH agonist +/− antiandrogen or orchiectomy	*T*‐score > = −2	ZOL	4 mg IV every 3/12	LS +4.7%, TH +1.6%	PBO	LS −2%, TH −2.1%	106 ZOL/109 PBO	12 months
Michaelson 2007 [32]	GnRH agonist	No BMD criteria. Excl if *T*‐score < 2.5	ZOL	4 mg IV once	LS +4.0%, +0.7% TH	PBO	LS −3.1%, −1.9% TH	22/grp	12 months
Ryan 2007 [33]	GnRH agonist or orchiectomy	No BMD criteria	ZOL	4 mg IV every 3/12	LS +4.9%, FN +0.9%	PBO	LS −2.2%, FN −3.2%	22 ZOL/20 PBO	12 months
Bhoompalam 2009 [40]	GnRH agonist +/− anti androgen or orchiectomy	Excl *T* < −2.0	ZOL	4 mg IV every 3/12	ADT < 1y: LS +5.12% ADT > 1y: LS +4.82%	PBO	ADT < 1y: −3.13% ADT > 1y +0.99%	ADT < 1y: 21 ZOL/23 PBO. ADT > =1y: 21 ZOL/19 PBO	12 months
Satoh 2009 [41]	GnRH agonist	No BMD criteria. Excl if *T* < −2.5	ZOL	4 mg IV once	LS +3.5%, FN +5.1%	PBO	LS −8.2%, FN −1.8%	20 ZOL/20 PBO	12 months
Casey 2010 [42]	Goserelin 10.8 mg	No BMD criteria	ZOL	4 mg IV every 3/12	LS +3.3% FN +1.8% TH +0.9%	PBO	LS −1.5% FN −1.7% TH −2.0%	91 ZOL/90 PBO	12 months
Kapoor 2011 [43]	GnRH agonist (leuprolide or goserelin)	*T*‐score < −1.0	ZOL	4 mg IV every 3/12	LS +7.93%, FN +5.05%	PBO	LS +0.82%, FN −0.48%	17 ZOL/14 PBO	12 months
Kachnic 2013 [44]	GnRH agonist	*T*‐score > −2.5	ZOL	4 mg IV every 6/12	LS +6%, L FN +3%, R FN +1%, L TH +1% R TH −2%	PBO	LS −5%, L FN −8%, R FN −6%, L TH −8% R TH −5%	50 ZOL/46 PBO	36 months
Lang 2013 [45]	GnRH agonist (leuprolide or goserelin)	No BMD criteria	ZOL	4 mg IV once pre‐ADT (Arm 1) vs. 4 mg IV once at 6 months (Arm 2) vs. 4 mg IV every 1/12 for 6/12 from 6 months (Arm 3)	LS +1.2% FN −1% (arm 1), LS +1.9% FN +0% (arm 2), LS +3.6% FN +1.8% (arm 3)	3 ZOL schedules	Difficult to interpret	14 (Arm 1), 15 (Arm 2), 13 (Arm 3)	24 months
Cheung 2020 [46]	GnRH agonist	No BMD criteria	ZOL	5 mg IV once	LS +8.4%, TH +4.4% by DXA. No change in volumetric BMD.	PBO	Not given	39 ZOL/37 PBO	24 months
Rodrigues 2007 [47]	GnRH agonist or orchiectomy	No BMD criteria	CLO/ZOL	CLO IV 1500 mg every 28 days/ZOL 4 mg every 1/12	CLO LS −0.72, ZOL LS −0.88 [absolute value]	PBO	LS −1.82 [absolute value]	39 CLO/24 ZOL/31 PBO	36 months
Smith 2009 [35]	GnRH agonist or orchiectomy	*T*‐score < −1.0 or osteoporotic fracture	Dmab	60 mg sc every 6/12	LS +5.6%	PBO	LS −1.0%	734/grp	24 months
Doria 2016 [48]	GnRH agonist +/− anti androgen or orchiectomy	*T*‐score < −1.0 and fragility fracture	Dmab	Dmab 60 mg sc every 6/12, ALN 70 mg/week	LS +5.6%	ALN	LS −1.1%	234 total	24 months
Yoshida 2020 [49]	GnRH agonist (leuprolide 11.25 mg or goserelin 11.3 mg)	No BMD criteria	Dmab/min	Dmab 60 mg sc every 24/52, MIN 50 mg every 1/12	Min: LS +2.5% FN +0.8% TH +0.9% Dmab LS +4.0% FN +2.9% TH +3.7%	PBO	LS −0.1% FN −0.7% TH −1.5%	36 min/36 Dmab/30 PBO	12 months
Kearns 2010 [50]	GnRH agonist	No BMD criteria	RIS/Es	RSD 30 mg/week vs. RSD 30 mg/week + EST 0.5 mg/day vs. EST 0.5 mg/day vs. PBO	FN RSD: −0.0104/Est +0.0068/RSD + EST +0.0190 [absolute value]	PBO	FN +0.096 [absolute values]	11 RSD/11 Est/11 RSD + EST/10 PBO	12 months
Taxel 2020 [51]	GnRH agonist	No BMD criteria	RIS	35 mg/week	LS +1.7%, FN +0.2%	PBO	LS −1.2%, FN −2.0%	20 RIS/20 PBO	6 months
Choo 2012 [52]	GnRH agonist	*T*‐score > −2.5	RIS	35 mg/week	LS −0.85%, FN −2.55%	PBO	LS −13.55%, FN −5.56%	52 RIS/52 PBO	24 months
Klotz 2012 [53]	Leuprolide	No BMD criteria	ALN	70 mg/week	LS +1.7%, FN +0.85%	PBO	LS −1.9%, FN −0.56%	84 ALN/102 PBO	12 months
Smith 2010 [54]	GnRH or orchiectomy	*T*‐score < −1	TOR	80 mg OD PO	LS +2.3%, TH +1.9%, FN +1.9%	PBO	Difficult to interpret	646 TOR/638 PBO	24 months
Morabito [55]	GnRH agonist + antiandrogen	*T*‐score < −2.5	NER	25 mg IM every 1/12	LS +0.73%, TH +0.81%, FN +0.679%	PBO	LS −4.9%, TH −1.9%, FN −1.2%	24 NER/24 PBO	12 months
Magno 2005 [56]	GnRH agonist +/− antiandrogen	*T*‐score < −2.5	NER	25 mg IM every 1/12	Group A: LS +1%, TH +0.9%, Group B: LS +2.5%, TH +1.5%	PBO	Group A: LS −4.9%, TH −1.9%, Group B: LS −1.5%, TH −1.0%	30 NER/30 PBO	12 months
Smith 2004 [57]	GnRH agonist	No BMD criteria	RAL	60 mg PO daily	LS +1.0%, TH +1.1%, FN +0.3%	PBO	LS −1.0%, TH +2.6%, FN −1.7%	19 RAL/22 PBO	12 months

Abbreviations: ADT, androgen deprivation therapy; ALN, alendronate; APD, pamidronate; BMD, bone mineral density; CLO, clodronic acid; Dmab, denosumab; Es, oestrogen; FN, femoral neck; GnRH, gonadotropin‐releasing hormone; IV, intravenous; LS, lumbar spine; Min, minodronic acid; NER, neridronic acid; OD, orally daily; PBO, placebo; RAL, raloxifene; RIS, risedronate; TH, total hip; TOR, toremifene; ZOL, zoledronic acid.

Due to lack of evidence, there are no widely established thresholds to guide the commencement of bone protective therapy for CTIBL. The NCCN recommends bone protective therapy if the DXA *T*‐score is ≤ −2.0 (i.e., two standard deviations below that of young normal controls) or the FRAX 10‐year fracture risk is ≥ 20% for major osteoporotic fracture or ≥ 3% for hip fracture [58]. However, these recommendations are derived from non‐ADT populations and do not incorporate the accelerated bone loss that occurs with ADT. The FRAX threshold may become more widely used given recent validation using data from 684 men with prostate cancer and 8606 men without prostate cancer in the Manitoba BMD Registry, where it reliably predicted incident fractures in men with prostate cancer [14].

A pragmatic approach in men being treated with ADT, as recently suggested in the Royal Australian College of General Practitioners and Healthy Bones Australia Guidelines, is that all men aged ≥ 70 years with a *T*‐score ≤ −2.5 (osteoporotic range) should commence anti‐resorptive therapy, and therapy should be considered if the FRAX 10‐year absolute risk of major osteoporotic fracture is ≥ 20% or that of hip fracture is ≥ 3%; or if the BMD *T*‐score is ≤ −2.0 [30]. Once initiated, bone protective therapy should be continued as long as ADT is prescribed, but this should be re‐evaluated after ADT cessation as the gonadal axis may recover in some men, with more rapid recovery reported in younger men (< 65 years) or in those with a shorter (< 24–30 months) duration of ADT [30].

We suggest that bone density is monitored by DXA every 12–24 months following ADT withdrawal and bone protective therapy recommenced if a new fragility fracture occurs and/or bone density declines. However, it is difficult to be overly prescriptive, as in practice, commencement of bone protective therapy is usually determined by local national funding restrictions. In particular, health insurance coverage and government subsidies will affect access to DXA scanning and bone protective therapy.

### 3.6 Anti‐Resorptive Agents

#### 3.6.1 Oral Bisphosphonates

Bisphosphonates bind to hydroxyapatite in the bone matrix and inhibit osteoclast activity, reducing bone resorption and stabilising bone density [59]. Due to widespread availability and low cost, oral bisphosphonates such as alendronate and risedronate are often used as first‐line bone protective therapy (Table 1). Guidelines suggest therapy is given for up to 5 years or following cessation of ADT, before reassessment [60].

##### 3.6.1.1 Alendronate

The Cancer and Osteoporosis Research with Alendronate and Leuprolide (CORAL) study found that in prostate cancer patients commencing ADT (*n* = 186), alendronate 70 mg once weekly for 1 year was associated with increased BMD of 1.7% and 0.7% at the lumbar spine and hip, respectively, compared to the placebo group, which lost 1.9% and 1.6% at these sites, respectively [53]. Another randomised study of prostate cancer patients (*n* = 112) receiving ADT for a median duration of 14 months found that 1 year of alendronate 70 mg once weekly was associated with increased BMD of 3.7% at the lumbar spine and 1.6% at the femoral neck compared to the placebo group, which had a fall in BMD of 1.4% and 0.7% at the corresponding sites, respectively [34].

##### 3.6.1.2 Risedronate

Risedronate also appeared to have a beneficial effect on BMD. An RCT of 104 patients (52 patients per arm) found that risedronate 35 mg once a week was associated with stable BMD at the lumbar spine of +0.85% at 2 years compared to a fall of 13.55% in the placebo group [52]. A smaller RCT in 40 men commencing ADT showed that risedronate at a dose of 35 mg once a week for 6 months increased lumbar spine BMD by 1.7% from baseline with no change at the hip, compared to the control group which had a fall in BMD of 2.0% and 2.2%, respectively [51].

Although these studies demonstrated the efficacy of oral bisphosphonates at increasing BMD, it is important to note that they were relatively small and of limited duration, and so were unable to provide conclusive evidence regarding reduction of fracture risk. Furthermore, while oral bisphosphonates are generally well tolerated, they may be associated with upper gastrointestinal side effects, such as oesophageal irritation and may have poor enteric absorption [53]. This may contribute to sub‐optimal patient medication adherence, which can undermine long‐term therapeutic effectiveness in real‐world clinical settings [61].

#### 3.6.2 Intravenous Bisphosphonates

Intravenous bisphosphonates such as zoledronic acid and pamidronate have excellent bioavailability, but may be limited by the need for IV access and potential renal toxicity. The latter is relevant for prostate cancer patients who are often older and may have kidney injury from renal tract obstruction.

##### 3.6.2.1 Zoledronic Acid

Zoledronic acid is the preferred IV bisphosphonate due to higher potency, short infusion time and need for infrequent administration. It is usually given annually for bone protection with reassessment after 3 years [60]. A study of 106 prostate cancer patients commencing ADT found that zoledronic acid 4 mg, 3‐monthly for 1 year was associated with increased lumbar spine BMD of 5.3% compared to a fall of 2.2% in the placebo group [31]. (The usual dose of zoledronic acid to treat osteoporosis is 5 mg IV once a year). A corresponding increase in BMD of 1.2% was also observed at the femoral neck compared to a loss of 2.1% in the placebo group [31]. Another RCT (*n* = 40 men) found that a single dose of IV zoledronic acid 4 mg increased BMD at 12 months at the lumbar spine by 4.0% and 0.7% at the total hip compared to a reduction of 3.1% and 1.9%, respectively, in the placebo group [32].

##### 3.6.2.2 Pamidronate

The use of pamidronate 60 mg IV every 12 for 48 weeks was associated with stable BMD compared to placebo, which was associated with a fall of 3.3% and 1.8% at the lumbar spine and total hip, respectively [37]. Again, due to small sample size, no studies have shown a beneficial effect on fracture risk. However, increased BMD should result in fewer fractures.

#### 3.6.3 Denosumab

Denosumab, a monoclonal antibody, inhibits receptor activator of nuclear factor kappa‐B ligand (RANKL), a key factor required for the formation, function and survival of osteoclasts—thereby preventing bone resorption [62]. It is given every 6 months by subcutaneous (sc) administration for the treatment of osteoporosis. In the setting of metastatic castrate‐resistant prostate cancer, guidelines recommend denosumab 120 mg every 4 weeks to reduce skeletal‐related events [63]. Denosumab should be used with caution in severe renal impairment and vitamin D deficiency, due to possible hypocalcaemia [64].

A large study randomised patients on ADT to denosumab 60 mg sc every 6 months or placebo (*n* = 734 patients per group). At 24 months, lumbar spine BMD increased by 5.6% in the denosumab group compared with a loss of 1.0% in the placebo group [35]. There was also an increase in BMD at the hip and distal forearm. Those who received denosumab had fewer new radiologic vertebral fractures at 36 months (1.5% compared to 3.9% with placebo, relative risk 0.38; 95% CI, 0.19–0.78; *p* = 0.006) [35]. This is the only intervention study to show a reduction in fracture risk in patients with prostate cancer on ADT. Of note, baseline lumbar spine BMD was not low in this study, with a mean *T*‐score of −0.3 in the denosumab group and −0.4 in the placebo group, respectively. Only ~15% of study participants had a *T*‐score of < −2.5 at any site. This suggests that earlier treatment might be beneficial. There are no published head‐to‐head studies comparing bone protective agents with fracture outcomes in this patient group.

The development of multiple vertebral fractures following denosumab discontinuation due to rebound bone resorption is well‐recognised [65]—especially in those with previous vertebral fractures [66]. Although definitive measures to prevent this remain uncertain, denosumab should either be continued long‐term or its cessation followed by another anti‐resorptive agent, for example, 12 months of an oral bisphosphonate (in patients treated with denosumab up to 2.5 years), or in those with longer use of denosumab, one or more zoledronic acid infusions [67, 68]. Despite the need for parenteral administration, denosumab is associated with improved medication adherence and patient preference [69].

#### 3.6.4 Estradiol

The ADT‐associated bone loss may not be directly related to reduction in serum testosterone, but rather due to reduction of estradiol [70]. In line with this, the randomised UK Prostate Adenocarcinoma TransCutaneous Hormones (PATCH) trial compared conventional ADT using gonadotropin‐releasing hormone (GnRH) analogues with estradiol patches, dosed to suppress gonadotrophins via negative feedback. While serum testosterone was reduced to castrate concentrations in both groups, patients treated with transdermal estradiol‐based ADT did not experience the BMD loss seen with GnRH analog‐based ADT [71]. Moreover, physiologic estradiol ‘add‐back’ to men undergoing conventional ADT with GnRH analogues reported that estradiol, compared to placebo, significantly increased lumbar spine and radius BMD and bone strength [72]. However, the efficacy of transdermal estradiol with respect to prostate cancer outcomes is still unknown, with current trials evaluating metastasis‐free survival in men with metastatic prostate cancer [73].

### 3.7 Bone Anabolic Agents

#### 3.7.1 Romosozumab

Romosozumab, a novel monoclonal antibody that binds sclerostin, an inhibitor of the bone‐catabolic *Wnt* signalling pathway, results in a marked BMD increase with a reduction in fracture risk in women with post‐menopausal osteoporosis [74, 75]. Unlike bisphosphonates and denosumab, which were discussed above and predominantly reduce bone resorption, and teriparatide (discussed later), which predominantly increases bone formation, romosozumab both increases bone formation and reduces bone resorption. This unique mechanism of action leads to a marked increase in BMD, greater than that seen with oral alendronate or teriparatide [74, 75].

However, the potent bone anabolic effect has raised concern about use in the setting of bone malignancy, and so there are no published data on its use in men with prostate cancer. The one study reporting romosozumab use in men with osteoporosis found that after 12 months, a significantly greater mean increase in lumbar spine BMD was seen in those on romosozumab compared with placebo (12.1% vs. 1.2%; *p* < 0.001) [76]. Lesser, but still significant, BMD increments were seen at the hip with romosozumab [76], leading the authors to conclude that it appeared effective and safe in men with osteoporosis. However, there are no intervention studies evaluating fracture outcomes in men.

Reassuringly, data from animal models indicated that sclerostin inhibition reduced bone metastases in a model of breast cancer [77] and prevented myeloma‐induced bone loss and reduced osteolytic bone lesions with no effect on tumor cell burden or activity [78, 79]. However, clinical studies in patients with an underlying malignancy are required before it can be considered for routine use in this clinical context.

#### 3.7.2 Parathyroid Hormone (PTH) Analogues

Teriparatide (recombinant human, rhPTH 1–34) and abaloparatide are PTH analogues which mimic the action of endogenous parathyroid hormone by stimulating osteoblast activity and promoting new bone formation [80]. They are highly effective in increasing BMD and reducing fracture risk in women with post‐menopausal osteoporosis [81, 82] and in men with osteoporosis [83]—although the study of abaloparatide in men was too small to show a reduction in fracture risk [84]. However, as for romosozumab, use of PTH analogues in men with prostate cancer undergoing ADT remains off‐label due to a theoretical risk of stimulating growth of occult bone metastases. Much of this concern arose from preclinical rodent studies, which found an increased risk of osteogenic sarcoma in rats treated with high dose teriparatide for near‐lifetime duration [85]. However, long‐term surveillance of human osteosarcoma cases found no relationship with teriparatide [86, 87].

Overall, we suggest that all men commencing ADT should undergo a baseline DXA scan for risk stratification. All men aged ≥ 70 years with a *T*‐score ≤ −2.5 (osteoporotic range) should commence anti‐resorptive therapy with denosumab or a bisphosphonate. Bone protective therapy should be considered if the FRAX 10‐year absolute risk of major osteoporotic fracture is ≥ 20% or that of hip fracture is ≥ 3%; or if the BMD *T*‐score is ≤ −2.0. Bone protective therapy should continue as long as ADT is prescribed. We suggest that bone density is monitored by DXA every 12–24 months following ADT withdrawal, and bone protective therapy recommenced if a new fragility fracture occurs and/or bone density declines.

### 3.8 Strategies to Improve Bone Health in Patients With Prostate Cancer

Despite international guidelines recommending a DXA scan prior to ADT commencement [15, 16, 17], this is often not done [19, 20, 21, 22, 23]. However, effective strategies may address this gap, for example, the Healthy Bones Program from Kaiser Permanente Southern California [88]. In this program, men with prostate cancer receiving leuprolide had a DXA scan, and those with *T*‐scores of −2.0 to −2.5 were advised on smoking cessation, regular exercise and an adequate oral calcium (1200 mg/day) and vitamin D intake (400–800 IU/day). Men with DXA *T*‐scores < −2.5 received bone protective therapy, mainly a bisphosphonate, with endocrinologist follow‐up. The programme was associated with a 72% reduction in hip fracture incidence compared to the control group who received usual care [88]. The ‘BoneRx’ healthy bone prescription tool was also effective at improving bone health in this patient group [89]. ‘BoneRx’ consisted of a package which prompted oncology treating healthcare professionals to refer for a DXA scan and provide advice regarding calcium and vitamin D supplementation and physical activity to patients with prostate cancer, reinforced by a patient educational booklet. Although post‐intervention BMD was not assessed, there was improvement in referrals for baseline DXA scans (34.7% vs. 59.5%, *p* < 0.0001), provision of bone health counselling (32.4% vs. 59.9%, *p* < 0.0001), use of vitamin D supplements (57% vs. 81%, *p* < 0.001), use of calcium supplements (39% vs. 61%, *p* < 0.001) and increased physical activity (*p* = 0.021) compared to pre‐intervention [89].

Other strategies to increase DXA use in this patient group include increasing physician awareness about the importance of bone health as part of cancer management and integration of DXA scans into routine oncology clinic visits. Establishment of a clinical pathway to routinely refer patients with low bone density to a bone health expert, for example, a rheumatologist or endocrinologist, may be of assistance. Greater patient education via consumer advocacy groups would also raise understanding of bone health management, including the need for DXA scanning in patients with prostate cancer.

## 4 Breast Cancer

### 4.1 Osteoporosis and Anti‐Oestrogen Therapy

Breast cancer is the commonest cancer among women, accounting for one in every eight cancer diagnoses, with an estimated 2.3 million new cases and 685,000 deaths worldwide in 2020 alone [90, 91]. Despite increasing incidence rates, mortality has declined over the past 30 years due to earlier detection and more targeted therapy [91, 92]. In developed countries, up to 80% of breast cancer is hormone receptor (oestrogen, ER or progesterone, PR) positive [93]. Endocrine therapy which lowers systemic oestrogen levels or blocks the effect of oestrogen on tumour cells is the mainstay of treatment [94]. While effective, it has deleterious effects on BMD. There are four main classes of endocrine therapy: (1) selective oestrogen receptor modulators (SERMs), like tamoxifen; (2) aromatase inhibitors (AI) such as letrozole, anastrozole and exemestane; (3) luteinizing hormone (LH)‐releasing hormone analogues like goserelin and leuprolide, usually used in combination with AIs and/or tamoxifen; and (4) the selective oestrogen receptor degrader, fulvestrant [95].

*Tamoxifen* is a SERM that competes with oestrogen in breast tissue, resulting in anti‐oestrogenic and anti‐tumour effects [96]. It has long been the standard of care for ER+ breast cancer. Tamoxifen has differing effects on bone depending on menopausal status, as it is a partial (i.e., less potent than estradiol itself) ER agonist in bone. In healthy women, BMD loss peaks at 2% per year around menopause for 5–10 years, which declines over time to ~1% per year [97, 98]. In post‐menopausal women, while tamoxifen reduces bone loss [99], this is not bone protective, as there were similar fracture rates in this population compared to healthy controls [100, 101, 102]. In pre‐menopausal women, tamoxifen induces bone loss of 1.44% per year [99], which was associated with increased fracture incidence compared to healthy controls [101, 103].

*Aromatase inhibitors (AIs)* reduce oestrogen production by inhibiting aromatase, the key enzyme in the conversion of androgens to oestrogen [104]. This reduces systemic oestrogen levels in post‐menopausal women because post‐menopausal women have quiescent ovaries and AI monotherapy suppresses the low residual levels of aromatase activity in peripheral tissues [104, 105]. As such, AI monotherapy is generally only used in post‐menopausal women. In pre‐menopausal women, where ovarian aromatase is active, AIs are not potent enough to suppress ovarian aromatase and, if used, need to be combined with ovarian suppression, either using GnRH analogues or ovariectomy.

While effective in both advanced and early‐stage breast cancer, AI therapy is associated with reduced BMD and increased fracture risk [106, 107, 108, 109]. A study of 249 post‐menopausal women with breast cancer found that those treated with AI had significantly lower BMD at the total hip and femoral neck compared to patients not on AI treatment [110]. Anastrozole decreased the median lumbar spine BMD and total hip BMD by 6.08% and 7.24%, respectively, from baseline over 5 years, compared to the tamoxifen group in which mean BMD increased by 2.77% and 0.74%, respectively [107]. A meta‐analysis of 30 RCTs that investigated osteoporotic fractures in breast cancer patients on AI therapy found an increased crude risk ratio of osteoporotic fractures of 1.35 (95% CI, 1.29–142), with a 1.18‐fold (95% CI, 1.02–1.35) and 1.84‐fold (95% CI, 1.36–2.49) increase in hip and vertebral fracture risk ratio, respectively [111]. However, in these oncologic trials, fractures were collected as adverse events rather than primary endpoints—likely underestimating fracture rates. In a fracture endpoint trial of 3420 post‐menopausal women [112], approximately 1 in 10 women had an incident clinical fracture within 3 years of AI initiation—comparable to fracture rates reported in untreated women 5–10 years older with established osteoporosis [113, 114]. This aligns with evidence that AIs accelerate the ageing process in women, potentially compounding bone loss and fracture risk [115].

*Luteinizing hormone‐releasing hormone (LHRH) analogues* block ovarian oestrogen production [116]. Pre‐menopausal women started on the LHRH analogue goserelin had a 5% loss of BMD over 2 years with partial recovery of +1.5% following cessation for 1 year [117]. In the same study, addition of tamoxifen appeared to counteract the demineralising effects of goserelin alone, resulting in less BMD decline (−1.5%) [117]. Addition of an LHRH agonist to AI therapy was associated with significant bone loss compared to LHRH + tamoxifen. In a cross‐sectional study, pre‐menopausal women with early breast cancer who received adjuvant AI + ovarian suppression for a mean of only 17 months had severe deterioration of bone microarchitecture with changes comparable to, or poorer than post‐menopausal controls 20 years older [118].

However, data on fracture outcomes remain limited as studies are often not powered for fracture reduction [119]. Use of LHRH + exemestane was associated with an increased incidence of osteoporosis by DXA criteria (38.6% vs. 25.2%) and increased fracture rates (6.8% vs. 5.2%) when compared to LHRH + tamoxifen over 5 years [120].

*Fulvestrant* is a selective ER down‐regulator that induces oestrogen degradation by competitively binding to the ER. While there are no large RCTs examining its effect on bone health, animal studies and small studies in humans suggest fulvestrant has no major adverse effects on bone health [121, 122].

### 4.2 Diagnosis of Cancer Treatment‐Induced Bone Loss (CTIBL)

The American Society of Clinical Oncology (ASCO) issued Guidelines in 2019 regarding osteoporosis management in survivors of adult cancers, including those with breast cancer on endocrine therapy [97]. They recommended that patients with non‐metastatic cancer with one or more risk factors for osteoporosis prescribed a drug that causes bone loss (such as AI) should have a baseline DXA scan with repeat BMD assessment by DXA every 1–2 years [98], as low BMD at AI initiation was predictive of future fracture [123]. Similar recommendations have been provided by ESMO [26], the Japanese Society of Bone and Mineral Research [124], and the Australian Endocrine, Bone and Mineral, Menopause and Oncologic Societies [125].

Despite this, baseline BMD testing among women with breast cancer on AI therapy remains low [126, 127, 128, 129]. A study of 9138 women older than 50 years from a commercial payer database in the United States, treated with AI for breast cancer between 2002 and 2008 found that only 41.6% underwent baseline DXA testing [129]. Another study of 19,585 US Medicare‐enrolled women older than 67 years found only 67.7% of women initiating adjuvant AI therapy underwent baseline BMD assessment [127]. However, these rates were higher than those in men commencing ADT for prostate cancer [19, 20, 21, 22]. Unfortunately, unlike for prostate cancer, the FRAX tool [13] has not been validated in women on AI treatment for breast cancer.

### 4.3 Prevention of CTIBL

Non‐pharmacologic strategies, such as regular exercise, and calcium and vitamin D supplementation, should be considered in breast cancer patients on endocrine therapy.

Despite the lack of evidence that exercise reduces fracture risk in this patient population, it is thought that enhancing muscle strength is important in falls prevention, and exercise has multiple benefits in women with breast cancer [130, 131]. As vitamin D deficiency is common in the general population—including in women with breast cancer [132, 133, 134], it is strongly advised that serum 25‐OH vitamin D levels be assessed prior to commencing AI therapy or upon detection of low BMD (osteopenia or osteoporosis) on baseline DXA. While there is scant evidence supporting the efficacy of calcium and vitamin D supplements for CTIBL in women with breast cancer [135], vitamin D supplementation for vitamin D‐deplete women may alleviate AI‐induced musculoskeletal symptoms [136, 137, 138]. As per advice for prostate cancer, consensus suggests a daily dietary intake of 1000–1200 mg of calcium and 800–1000 IU of vitamin D for women undergoing AI therapy is reasonable [135, 139, 140, 141, 142].

### 4.4 Bone Protective Agents

The ASCO Guidelines have suggested that pre‐menopausal women receiving LHRH agonists resulting in ovarian suppression and post‐menopausal women on AIs should be offered bone protective therapy if the DXA *T*‐score is < −2.5 at any site, or in those at increased risk of osteoporotic fracture based on clinical assessment or risk assessment tools, such as FRAX [98]. An Australian Position Statement [141] recommended the addition of anti‐resorptive therapy in those with a fragility fracture, including subclinical vertebral fracture, at any age, or in women ≥ 70 years of age with a DXA *T*‐score ≤ −2.5 [141]. For women on AI therapy not fulfilling this criteria, the recommendation is to consider anti‐resorptive therapy in the following circumstances: if the BMD *T*‐score is < −2.0 at any site, the presence of ≥ 2 fracture risk factors, if there is a ≥ 5% and/or ≥ 0.05 g/cm^2^ decrease in BMD in 1 year, or if the FRAX 10‐year risk for major fracture is > 20% or hip fracture is > 3% [141]. For pre‐menopausal women, this should change to a *Z*‐score of < −2.0 (DXA BMD more than two standard deviations lower than age‐ and sex‐matched controls), or a *Z*‐score of < −1.0 with an annual decrease in BMD of 5% [141]. However, as for prostate cancer, local governmental funding may determine access to bone protective therapy.

### 4.5 Anti‐Resorptive Agents

#### 4.5.1 Oral Bisphosphonates

Oral bisphosphonates, including ibandronate, alendronate and risedronate, have demonstrated efficacy in reducing bone loss from AI therapy in this patient population (Table 2).

**TABLE 2:** Randomised controlled trials of bone protective therapy in patients with breast cancer treated with anti‐oestrogen therapy.

Reference number	Breast cancer therapy	Inclusion criteria	Drug	Dose	BMD change in intervention group	Controls	BMD change in controls	Patient numbers	Timepoint of primary outcome
Gnant 2007 [143]	Tamoxifen + goserelin	Pre‐menopausal	ZOL	4 mg q6m	LS −2.6%, TH +0.6%	No treatment	LS −17.4%, −11.6%	ZOL 95, Control 79	12 months
Gnant 2008 [119]	Anastrozole + goserelin	Nil BMD, but patient with osteoporosis excluded	ZOL	4 mg q6m	LS +0.4%, FN +0.8%	No treatment	LS −11.3%, FN −7.3%	ZOL 205, Control 199	36 months
Lee 2011 [144]	Anastrozole or letrozole	Postmenopausal hormone receptor positive breast cancer treated with AI	ZOL	4 mg q3m or q6m	LS +3.85%, TH +1.8%	No treatment	LS −8.17%, TH −6.82%	ZOL 48/Control 59	36 months
Nuzzo 2012 [145]	Letrozole	ER+ breast cancer	ZOL	4 mg q6m	LS +0.02 SD	No treatment	LS −0.57	ZOL 154, Control 149	12 months
Kalder 2015 [146]	(Neo) adjuvant CT or endocrine therapy	*T*‐score < −2.5 excluded	ZOL	4 mg q3m	LS +3.1%	PBO	LS −6.4%	ZOL 34, PBO 36	24 months
Sun 2016 [147]	Letrozole	*T*‐score < −2.0	ZOL	4 mg q6m	LS +3.7%, TH +2.1%	PBO	LS −1.85%, TH −1.05%	ZOL 60, PBO 60	12 months
Brufsky 2009 [148]	Letrozole	*T*‐score between −1.0 and −2.5 at LS and TH	(Early) ZOL	4 mg q6m	LS +3.853%, TH +1.676%	(Delayed) ZOL	LS −2.990%, TH − 3.463%	Early ZOL 301, Delayed ZOL 301	36 months
Hines 2009 [149]	Letrozole 2.5 mg daily	Postmenopausal women, *T*‐score ≥ −2	(Early) ZOL	4 mg q6m	LS +4.94%, FN +3.36%, TH +1.22%	(Delayed) ZOL	LS −2.28%, FN +0.54%, TH −3.34%	Early ZOL 274, Delayed ZOL 277	24 months
Brundred 2008 [150]	Letrozole 2.5 mg daily	Postmenopausal women, *T*‐score ≥ −2	(Early) ZOL	4 mg q6m	LS −0.2%, TH +0.07%	(Delayed) ZOL	LS −5.5%, TH −3.4%	Early ZOL 532, Delayed ZOL 533	12 months
Brufsky 2007 [151]	Letrozole 2.5 mg daily	*T*‐score between −1.0 and −2.5 at LS and TH	(Early) ZOL	4 mg q6m	LS +1.96%, TH +1.16%	(Delayed) ZOL	LS −2.36%, TH −1.88%	Early ZOL 301, Delayed ZOL 301	12 months
Eidtmann 2010 [152]	Letrozole 2.5 mg daily	Postmenopausal women, *T*‐score ≥ −2	(Early) ZOL	4 mg q6m	LS +4.39%	(Delayed) ZOL	LS −4.9%	Early ZOL 532, Delayed ZOL 533	36 months
Brufsky 2011 [153]	Letrozole 2.5 mg daily	*T*‐score between −1.0 and −2.5 at LS and TH	(Early) ZOL	4 mg q6m	LS +6.19%, TH +2.58%	(Delayed) ZOL	LS −2.41%, TH −4.12%	Early ZOL 301, Delayed ZOL 301	60 months
Llombart 2012 [154]	Aromatase inhibitors	*T*‐score between −1.0 and −2.5 at LS and TH	(Early) ZOL	4 mg q6m	LS +2.7%, TH +1.7%	(Delayed) ZOL	LS −2.7%, −1.6%	Early ZOL 263, Delayed ZOL 264	12 months
Takahashi 2012 [155]	Letrozole	Post‐menopausal women	(Early) ZOL	4 mg q6m	LS +4.9%, TH +4.4%	(Delayed) ZOL	LS −2.0%, TH −2.4%	Early ZOL 94, Delayed ZOL 95	12 months
Coleman 2013 [156]	Letrozole	*T*‐score between −1.0 and −2.5 at LS and TH	(Early) ZOL	4 mg q6m	LS +4.3%	(Delayed) ZOL	LS −5.4%	Early ZOL 532, Delayed ZOL 533	60 months
Wagner‐Johnson 2015 [157]	Letrozole 2.5 mg daily	Postmenopausal women, *T*‐score ≥ −2	(Early) Zol	4 mg q6m	LS +0.58	(Delayed) ZOL	LS −0.24	Early ZOL 274, Delayed ZOL 277	36 months
Ellis 2008 [158]	Aromatase inhibitors	*T*‐score between −1.0 and −2.5 at LS and TH	Dmab	60 mg q6m	LS +4.6%, TF +3.1%	PBO	LS −0.7%, TH −0.7%	Dmab 125, Control 125 PBO	12 months
Gnant 2015 [112]	Aromatase inhibitors	Post‐menopausal women	Dmab	60 mg q6m	LS +7.3%, TH +4.6%	PBO	LS −2.75%, TH −3.32%	Dmab 1711, PBO 1709	36 months
Van Poznak 2010 [159]	Anastrozole	*T*‐score between −1.0 and −2.5 at LS and TH	RIS	35 mg weekly	LS +2.2%, TF +1.8%	No treatment	LS −1.8%, TH −1.1%	RIS 77, PBO 77	24 months
Markopoulos 2010 [160]	Anastrozole	*T*‐score between −1.0 and −2.5 at LS and TH	RIS	35 mg weekly	LS +5.7%, TH +1.6%	No treatment	LS −1.5%, TH −3.9%	RSD 33, No treatment 37	24 months
Sestak 2014 [161]	Anastrozole	*T*‐score between −1.0 and −2.5 at LS and TH	RIS	35 mg weekly	LS +1.1%, TH −0.7%	PBO	LS −2.6%, TH −3.5%	RSD 136, PBO 123	36 months
Greenspan 2015 [162]	Aromatase inhibitors	*T*‐score between −1.0 and −2.5 at LS and TH	RIS	35 mg weekly	LS +2.27%, TH +0.556%	PBO	LS −1.1735%, TH −2.748%	RSD 55, PBO 54	24 months
Sestak 2019 [163]	Anastrozole	*T*‐score between −1.0 and −2.5 at LS and TH	RIS	35 mg weekly	LS −0.4%, TH −2.5%	PBO	LS −4.2%, TH −3.8%	RSD 136, PBO 123	60 months
Rhee 2013 [164]	Aromatase inhibitors	Postmenopausal	ALN	5 mg alendronate +0.5 ug of calcitriol	LS −0.5%, TH −0.5%	No treatment	LS −3.5%, TH −1.3%	ALN 49, Control 49	6 months
Livi 2019 [165]	Aromatase inhibitors	*T*‐score between −1.0 and −2.5 at LS and TH	IBA	150 mg monthly	LS +0.35%, TH +0.28%	PBO	LS −0.13%, TH −0.24%	IBA 89, PBO 82	24 months
Saarto 1997 [166]	Tamoxifen	Post‐menopausal women	CLO	1600 mg daily	LS +2.5%, FN +4.2%	No treatment	LS −1.5%, FN +2.0%	CLO 14/No treatment 11	24 months

Abbreviations: ALN, alendronate; BMD, bone mineral density; CLO, clodronic acid; Dmab, denosumab; FN, femoral neck; IBA, ibandronic acid; IV, intravenous; LS, lumbar spine; OD, orally daily; PBO, placebo; RIS, risedronate; TH, total hip; ZOL, zoledronic acid.

Two notable trials have explored the impact of monthly ibandronate in this context. The Impact of Monthly Oral Ibandronate on BMD loss for Osteopenic Post‐menopausal Patients Treated with Adjuvant AI (BONADIUV) study found that patients administered ibandronate 150 mg once every 28 days had a mean *T*‐score increase of +0.28 at the total hip and +0.35 at the lumbar spine, compared to placebo (−0.09 and −0.24, respectively) [165]. The Effect of Oral Ibandronate on Anastrozole‐Induced Bone Loss (ARIBON) study, involving osteopenic breast cancer patients on anastrozole monotherapy, reported similar beneficial effects on bone [167]. Osteoporotic post‐menopausal women with breast cancer on anastrozole demonstrated a significant increase in BMD at the spine (15.6%) and hip (5.6%) at 3 years with alendronate 70 mg once weekly [168]. In the Study of Anastrozole with the Bisphosphonate Risedronate (SABRE) trial, oral risedronate 35 mg once weekly was assessed in post‐menopausal women with hormone receptor‐positive early breast cancer undergoing adjuvant anastrozole therapy, stratified by fracture risk. Higher‐risk patients treated with risedronate had a significant increase in lumbar spine and total hip BMD from baseline (3.0% and 2.0%, respectively). Similarly, moderate‐risk patients on risedronate had a significant increase in BMD compared to those on placebo (2.2% vs. −1.8% for spine and 1.8% vs. −1.1% for total hip) [159]. Comparable results were observed in the Arimidex Bone Mass Index and Oral Bisphosphonates (ARBI) prospective clinical trial [160]. Moreover, risedronate use in women with low bone mass undergoing AI therapy effectively suppressed serum markers of bone turnover at 12 months, with greater suppression correlated with spinal BMD increase at 24 months [162]. Guidelines recommend the use of an oral bisphosphonate for 3–5 years [26], with reassessment 12 months after initiation. Nevertheless, most studies were limited by small sample size, meaning that none demonstrated a reduction in fractures.

#### 4.5.2 Intravenous Bisphosphonates

Use of parenteral bisphosphonates, particularly zoledronic acid, has been shown to effectively reduce bone loss associated with anti‐oestrogen treatment in women with breast cancer (Table 2) [119, 148, 149, 154, 155, 156, 157, 169, 170]. In the bone density sub‐study of the Austrian Breast and Colorectal Cancer Study Group (ABCSG)‐*12* trial, zoledronic acid 4 mg IV, 6‐monthly prevented bone loss induced by combination LHRH agonist and tamoxifen, as well as in pre‐menopausal breast cancer patients on LHRH agonist and anastrozole [119]. Among post‐menopausal women, three independent yet similarly designed trials, namely the Zometa‐Femara Adjuvant Synergy Trials‐Z‐FAST [148], ZO‐FAST [156] and E‐ZO‐FAST [154] investigated early zoledronic acid initiation in women receiving letrozole compared to delayed treatment. In all three trials, early zoledronic acid averted bone loss associated with AI therapy. At 5‐years, in the Z‐FAST trial, early zoledronic acid (4 mg IV, 6‐monthly) resulted in an 8.9% difference in lumbar spine BMD between the early and delayed treatment groups [148]. The ‘Does Adjuvant Zoledronate redUce REcurrence in early breast cancer AZURE)?’ trial investigated the efficacy of zoledronic acid in preventing fractures over a 5‐year period in patients with stage II/III breast cancer undergoing neoadjuvant/adjuvant chemotherapy and/or endocrine therapy. The use of zoledronic acid 4 mg IV in a pre‐defined 5‐year treatment regimen (every 3–4 weeks for six doses, 3‐monthly for eight doses and then 6‐monthly for a further five doses) was associated with a decrease in 5‐year fracture risk from 5.9% to 3.8% and a significantly prolonged time‐to‐first fracture (HR 0.69, 95% CI 0.53–0.90) [171]. Fracture prevention benefits were consistent across both pre‐ and post‐menopausal subgroups [171]. Based on the above data, guidelines recommend IV zoledronic acid 6‐monthly for at least 36 months (~ six doses) [26], with further doses as required.

#### 4.5.3 Denosumab

The Hormone Ablation Bone Loss Trial in Breast Cancer (HALT‐BC) trial examined 252 women with early breast cancer on AI therapy randomised to placebo or denosumab (60 mg sc, 6‐monthly). After 24 months, BMD increased by 7.6% at the lumbar spine compared to placebo (+6.0% vs. −1.6%, respectively), with increases also seen at the total hip and femoral neck [158]. This result was confirmed in the larger ABCSG‐18 trial—a double‐blind, placebo‐controlled trial of 3420 women with early breast cancer on AI therapy [112]. At 36 months, those in the denosumab group had an increase in BMD of 10% at the lumbar spine, 7.9% at the total hip and 6.5% at the femoral neck. The increase in BMD correlated with halving of fracture rates (5.0% vs. 9.6% for placebo) and delayed time‐to‐first clinical fracture compared to placebo (HR 0.5, 95% CI 0.39–0.65) [119]. Noticeable in this fracture outcome trial was the high incidence of clinical fractures (9.6%) in the placebo group, even though at baseline most women had normal BMD (55% with lumbar spine *T*‐scores of > −1.0). This study raised the question of whether guideline recommendations should consider earlier anti‐resorptive treatment. Long‐term follow‐up of the ABCSG‐18 trial showed that even 11 years after randomisation, there remained a difference in fracture outcomes, with only 15.9% of patients in the denosumab group experiencing a clinical fracture, compared to 19.2% of those in the placebo group (HR 0.76, 95% CI 0.63 to 0.92). There was no increase in rebound fracture risk following denosumab cessation (4.6% in denosumab vs. 5.1% in the placebo arm 2.5 years after last randomised treatment) [172]. Despite this, the duration of denosumab therapy must be carefully considered given the increased risk of vertebral fracture after cessation.

A similar delay in incident clinical fracture was also seen in the D‐Care trial of 4509 women with high‐risk early breast cancer in both pre‐ and post‐menopausal subgroups [173]. While this study did not reach its primary endpoint (improvement in bone metastasis‐free survival), denosumab use appeared to have bone protective effects with a reduction in HRs for time‐to‐first on‐study fracture (HR 0.76, 95% CI 0.63–0.92) and time‐to‐first on‐study skeletal‐related event (HR 0.52, 95% CI 0.35–0.78) [173]. The benefit of denosumab has recently been extended to pre‐menopausal women initiating estradiol depletion therapy. While women assigned to placebo lost up to 10% of bone within 12 months of estradiol depletion therapy initiation, denosumab prevented this bone loss and suppressed bone remodelling markers [174].

#### 4.5.4 Bone Anabolic Agents

Use of romosozumab and PTH analogues in the setting of breast cancer remains unexplored for the reasons outlined above for prostate cancer.

### 4.6 Strategies to Improve Bone Health in Breast Cancer

Despite multiple guidelines recommending baseline BMD testing in all women with breast cancer on endocrine therapy, testing rates remain sub‐optimal [126, 127, 128, 129]. A UK study showed that an education campaign targeting clinicians, including posters summarising current guidelines and discussing the need for DXA scans, was associated with improvement in referral for baseline DXA scans from 38% to over 90% at 1 year [175]. Provision of educational materials has also been shown to increase BMD testing in this patient population [176]. Comprehensive bone health programs that included counselling, exercise, nutritional advice, vitamin D supplements and, when required, pharmacological therapy with bisphosphonates have been shown to stabilise and/or improve BMD in post‐menopausal women on AI therapy [177]. Further research into such interventions/models of care, like that of BoneRx in prostate cancer [89], is required.

Overall, all women with breast cancer prescribed a drug that induces bone loss and who have at least one risk factor for osteoporosis should undergo a baseline DXA scan, with repeat assessment every 1–2 years. Women with osteoporosis (DXA *T*‐score ≤ −2.5 at any site), or those at increased risk of fracture, should be initiated on bone protective therapy with denosumab or a bisphosphonate. Bone protective therapy should also be considered for women with osteopenia (*T*‐score between −1 and −2.5) and more than two fracture risk factors and/or evidence of significant BMD loss on treatment.

## 5 Conclusion

The excellent long‐term prognosis of patients with prostate and breast cancer is a tribute to enormous advances in early diagnosis, treatment and multi‐disciplinary care by the oncology and surgical community. However, the extended survival of these patients with the use of more potent endocrine therapies means bone health is an increasingly important part of cancer survivorship. Radiation and medical oncologists, urologists, bone health experts, general practitioners, healthcare professional bodies and bone health and cancer consumer organisations should increase awareness of the potential adverse effect of hormone therapy on bone health. While this should never delay cancer treatment, bone health should be part of routine care for men and women receiving hormone therapy. Streamlined referral processes, for example, using clinical pathways, may facilitate timely DXA assessment and, if required, early initiation of bone protective therapy—usually a bisphosphonate or denosumab combined with calcium and vitamin D. Close collaboration with a bone health expert is recommended.

## Author Contributions

**Ian Liang:** data curation (equal), writing – original draft (lead). **Sarah Brennan:** data curation (equal), writing – original draft (lead). **Christian Girgis:** supervision (equal), writing – original draft (supporting), writing – review and editing (lead). **Amy Hayden:** writing – review and editing (equal). **Tania Moujaber:** writing – review and editing (equal). **Sandra Turner:** writing – review and editing (equal). **Anuradha Vasista:** writing – review and editing (equal). **Mathis Grossmann:** writing – review and editing (equal). **Peter K. K. Wong:** conceptualization (lead), supervision (lead), visualization (equal), writing – original draft (equal), writing – review and editing (lead).

## Conflicts of Interest

C.G. has received advisory board consultancy fees from Sandoz and a speaker honorarium from Gedeon Rechter. T.M. has received advisory board consultancy fees from Merck, Pfizer and BMS, and speaker honoraria from Merck, Pfizer and Amgen. P.K.K.W. has been a site investigator for a Phase IV Amgen trial.

## Data Availability

The data that support the findings of this study are available from the corresponding author upon reasonable request.
